# A COVID-19 Patient Presenting with Pneumothorax

**DOI:** 10.1155/2021/6652819

**Published:** 2021-04-14

**Authors:** Seyyedeh Narjes Abootalebi, Eslam Shorafa, Alireza Abbaspour, Seyed Sina Dehghani, Mehdi Forooghi

**Affiliations:** ^1^Department of Pediatrics, School of Medicine, Shiraz University of Medical Sciences, Shiraz, Iran; ^2^School of Medicine, Shiraz University of Medical Sciences, Shiraz, Iran; ^3^Department of Pediatric Surgery, Namazee Hospital, Shiraz University of Medical Sciences, Shiraz, Iran

## Abstract

Severe acute respiratory syndrome coronavirus 2 (SARS-CoV-2) is a novel virus that has affected millions of individuals across the world. It was officially declared as a pandemic on March 11, 2020. Although most patients with COVID-19 manifest as viral pneumonia characterized by symptoms such as fever, dyspnea, and cough, atypical presentations such as acute respiratory distress syndrome (ARDS) and acute kidney or cardiac injury have been reported amongst COVID-19 patients. Bilateral and peripheral ground-glass and opacities are the hallmarks of COVID-19 infection on imaging exams. Herein, we aim to describe a COVID-19 patient who presented with shortness of breath, neck pain, chest pain, and bilateral pneumothorax in his imaging exam.

## 1. Introduction

Severe acute respiratory syndrome coronavirus 2 (SARS-CoV-2) is a novel virus that has affected millions of individuals across the world. Coronavirus disease 2019 (COVID-19) was officially declared as a pandemic on March 11, 2020, with the increase in the number of cases and countries affected and the sustained risk of further global spread [1–5]. This disease is most frequently manifested as viral pneumonia characterized by symptoms such as fever, dyspnea, and cough. Aside from common symptoms, severe secondary complications such as acute respiratory distress syndrome (ARDS) and acute kidney or cardiac injury were reported [6]. Chest imaging is abnormal in a great number of patients, even among asymptomatic and mild patients. The hallmarks of COVID-19 infection on imaging exams are bilateral and peripheral ground-glass and opacities [7, 8]. Herein, we present a positive COVID-19 PCR patient referred to Namazi Hospital, Shiraz, Iran, who had not any potential signs of COVID-19 in his chest CT such as peripheral distribution or mixed ground-glass opacity. However, there was evidence of bilateral pneumothorax in his imaging exam.

## 2. Case Presentation

**Figure 1:**
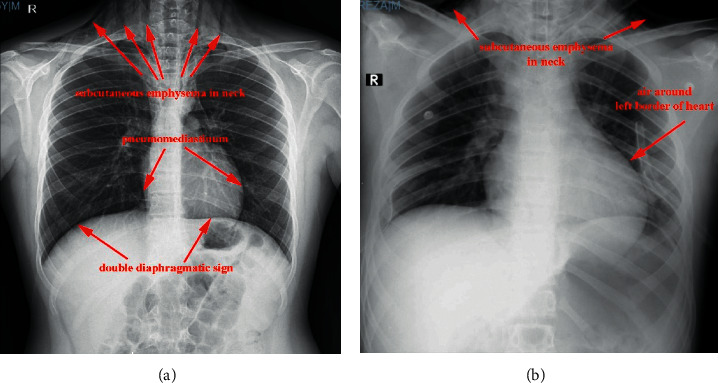
Chest X-ray revealing double diaphragmatic sign, air around the left border of the heart, and subcutaneous emphysema in the neck associated with pneumomediastinum.

**Figure 2:**
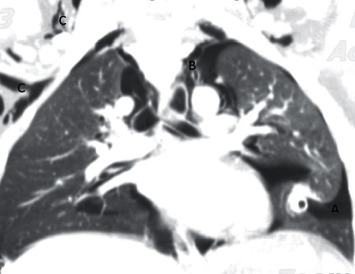
Chest computer tomography revealing significant emphysematous. A: pneumothorax; B: pneumomediastinum; and C: subcutaneous emphysema.

A 15-year-old boy not known to have any chronic medical condition was brought to the emergency department (ED) of Namazi Hospital with shortness of breath, neck pain, and chest pain on the last day before admission. The patient had no history of cough or any contact to infected patients. On initial physical examination in ED, his heart rate was 120 beats/min and the temperature was 37°C. He had a respiratory rate of 40 per minute. The peripheral O_2_ saturation was 87%. The patient's height and weight were 167 cm and 64 kg, respectively. Blood gas analysis (arterial) at admission showed a pH of 7.39, PCO_2_ of 33.2 mmHg, PO_2_ of 52.8 mmHg, and HCO_3_ of 20.5 mmHg. Due to his shortness of breath, chest X-ray (Figure 1) was done for him, in which evidence of double diaphragmatic sign was seen suggestive of pneumothorax. Also, evidence of pneumomediastinum was seen associated with subcutaneous emphysema in the neck. Another chest X-ray revealed evidence of air around the left border of the heart, which is the most common presentation of pneumomediastinum in the chest X-ray. Due to his decreased level of O_2_ saturation and symptomatic pneumothorax, he was referred to the operating room for intubation and insertion of a bilateral chest tube in the posterior side of the chest. Spiral CT scan of neck, chest, and mediastinum (Figure 2) was performed for him, which revealed a significant emphysematous change in the neck. The upper chest wall and the right axillary area were associated with bilateral pneumothorax, airways were grossly intact, and both lungs' field was cleared. It was not in favor of COVID-19, but due to the pandemic, the PCR of COVID-19 was done on samples of pharynx and nasopharynx of the patient which turned positive. Considering the patient's condition, he was admitted to the surgical pediatric intensive care unit (SPICU).  Table 1 shows the laboratory data of the patient.

## 3. Discussion

Pneumothorax is the accumulation of extrapulmonary air within the chest, most commonly due to leakage of air from the lung. Secondary spontaneous pneumothorax is arising as a complication of an underlying lung disorder without any trauma [9]. In this case report, the patient did not have any preexisting pulmonary conditions; it seems structural lung injury caused pneumothorax following COVID-19 pneumonia. However, it has not been seen in chest CT. Although the mechanism of the injury is not completely understood, according to the significant emphysematous change in his chest CT, maybe diffuse alveolar injury caused by COVID-19 can lead to alveolar rupture, which can cause air leakage and interstitial emphysema [10]. It is necessary to know if this pneumothorax was secondary to this virus or it was just an accidental coinfection between this pneumothorax and COVID-19.

No written consent has been obtained from the patients as there are no patient identifiable data included in this case report.

## 4. Conclusion

To the best of our knowledge, up till now, in other published case reports which were about association of pneumothorax and COVID-19, the patients had both pneumothorax and lung involvement simultaneously. This case report is the first published report of a COVID-19 patient who presented with pneumothorax without lung parenchymal involvement.

It seems that due to COVID-19 pandemic, it is reasonable to do COVID-19 PCR test on samples of pharynx and nasopharynx of the suspicious patient, who presented with irrelevant symptoms of coronavirus.

## Figures and Tables

**Table 1:** Paraclinical findings of the patient during her course of hospitalization.

Lab data	On admission	Before discharge	Normal reference values
WBC (/mm^3^)	15100	6400	4500–13000
Neutrophil percentage	87.7	70.9	45–75
Lymphocyte percentage	6.9	21	20–45
Hb (gm/dL)	13.2	12.6	13–16
MCV (fL)	84	84.6	79–98
Platelets (/mm^3^)	311,000	300,000	150–450
PT (sec)	18	15.6	12–14
INR (index)	1.33	1.16	0.9–1
PTT (sec)	31.9	32.2	25–35
BUN (mg/dL)	17	10	8–20
Cr (mg)	0.8	0.8	0.8–1.3
Na (mEq/L)	146	143	136–145
K (mEq/L)	4.4	4.4	3.5–5.5
Ca (mg/dL)	10	—	8.6–10.3
Mg (mg/dL)	2.1	—	1.7–2.2
Albumin (g/dl)	4.5	—	3.8–4.2
LDH (U/L)	356	—	<480
CRP (mg/L)	5	—	<6
Troponin (ng/mL)	<1.5	—	<1.5
Blood culture		No growth	
Urine culture		No growth	
